# Pharmacological Treatments and Adverse Reactions Following Snake Antivenom Therapy: A Collaborative Study by Healthcare Professionals in the Southernmost Region of Thailand

**DOI:** 10.3390/toxins18030139

**Published:** 2026-03-12

**Authors:** Panuwat Promsorn, Wittawat Chantkran, Musleeha Chesor, Janeyuth Chaisakul

**Affiliations:** 1 Galyani Vadhana Karun Hospital, Faculty of Medicine, Princess of Naradhiwas University, Narathiwat 96000, Thailand; panuwat.p@pnu.ac.th; 2 Department of Pathology, Phramongkutklao College of Medicine, Bangkok 10400, Thailand; c.wittawat@pcm.ac.th; 3 Faculty of Medicine, Princess of Naradhiwas University, Narathiwat 96000, Thailand; musleeha.c@pnu.ac.th; 4 Department of Pharmacology, Phramongkutklao College of Medicine, 317 Ratchawithi Road, Ratchathewi District, Bangkok 10400, Thailand

**Keywords:** venom, envenomation, snakebite, adverse reaction, epinephrine, anaphylaxis

## Abstract

The administration of specific immunoglobulin G-based antivenoms is a key strategy for treating snakebite envenoming victims. However, serious adverse reactions, such as anaphylaxis or serum sickness, are frequently observed following such administration. In addition, inflammation associated with delayed wound healing considerably drives the irrational use of antibiotics or anti-inflammatory agents, which may be linked to adverse reactions following antivenom treatment. In this study, we evaluated the factors contributing to adverse effects following the administration of snake antivenom, especially pharmacological treatment and premedication intended to prevent adverse reactions. Our retrospective study was conducted by healthcare professionals in Narathiwat, the southernmost province in Thailand, and it involved 980 patients confirmed to have been snakebitten from 2016 to 2021. Of these cases, 513 were treated with antivenom. Prevalence rates and 95% confidence intervals were calculated, and univariate and multivariate analyses were performed to determine the correlation between adverse reactions and medications. Following antivenom administration, the majority of the patients exhibited no adverse reactions (86.7%). Nevertheless, skin rash, itching, wheezing, angioedema, chest tightness, and fever were observed in 13.3% of those receiving snake antivenom. After the administration of antivenom for Malayan pit viper bite, adverse reactions occurred in 11.7% of the sample, especially among referral patients (*p* < 0.001). Epinephrine and antihistamines were prescribed as prevention and treatment for hypersensitivity due to antivenom administration. Antibiotics, Non-steroidal Anti-inflammatory drugs (NSAIDs), and acetaminophen were not associated with antivenom-induced adverse reactions. Interestingly, tramadol and antihistamines significantly reduced the occurrence of adverse reactions after antivenom administration (*p* < 0.05). Well-trained staff, close monitoring alongside resuscitation equipment and medications that can minimise the severity of anaphylactic reactions must be promptly available whenever antivenom is administered.

## 1. Introduction

Snakebite envenoming is a neglected tropical disease in the rural areas of low- and middle-income countries, especially sub-Saharan Africa, South America, South Asia, and Southeast Asia. This health problem results in a high mortality rate and causes physical and psychological disorders, and requires a One Health approach [1]. A report released by the World Health Organization (WHO) indicated that there are an estimated 2 million snakebite envenoming cases in Asia annually and that there are around 600,000 snakebite victims who need effective treatment in Africa each year [2]. These cases are an economic burden for governments in countries where health systems are weak and medical resources are sparse. The WHO intends to halve the global mortality and morbidity burdens of snakebites by 2030 [3,4].

In Thailand, snakebite incidence is anticipated to be approximately 500 to 5000 bites per year, but this comes with an admirably low mortality rate of less than 0.05% [5]. Especially in the southern region of the country, the leading cause of envenomation is the Malayan pit viper (*Calloselasma rhodostoma*), followed by green pit vipers (*Trimeresurus albolabris* and *Cryptelytrops macrops*) and cobras (*Naja kaouthia* and *N. siamensis*) at 37% and 16% incidence, respectively. Russell’s viper (*Daboia siamensis*) and Malayan krait (*Bungarus candidus*), which are categorised as venomous snakes of category 1, also cause numerous snakebites, resulting in high levels of morbidity and mortality in Thailand.

The clinical outcomes observed following snakebite envenoming include the presence of two needlestick fang marks; local effects, such as skin blistering, ecchymoses, haemorrhagic blebs, wound inflammation and tissue necrosis; and systemic effects, such as venom-induced consumption coagulopathy that causes abnormal blood clotting and systemic bleeding following viper envenomation or paralysis of skeletal muscles resulting in respiratory failure due to neurotoxic snake envenoming (i.e., by cobras and kraits) [6,7]. These local and systemic toxicities may be associated with serious disability and fatality if effective treatment or snake antivenom administration is delayed [8]. Early antivenom administration is the only effective treatment for snakebite envenoming. Snake antivenoms are immunoglobulin (IgG)-based products isolated from hyperimmune animal plasma/serum.

In Southeast Asia, the Queen Saovabha Memorial Institute (QSMI) is an important manufacturer of snake antivenoms, with the institution producing seven monospecific antivenoms (i.e., those for *N. kaouthia*, *Ophiophagus hannah*, *B. candidus*, *B. fasciatus*, *T. albolabris*, *D. siamensis*, *C. rhodostoma*) and two polyvalent snake antivenoms. The indications that warrant hematotoxic snake antivenom administration include a venous clotting time (VCT) > 20 min, unclotted 20 min whole blood clotting time (20WBCT), an international normalised ratio (INR) > 1.2, a platelet count < 50 × 10^3^ /μL, systemic bleeding, and impending compartment syndrome [5]. The criteria for the administration of antivenom for systemic envenomation following neurotoxic, cardiotoxic, and nephrotoxic snakebites are the presence of skeletal muscle paralysis (i.e., external ophthalmoplegia or ptosis), cardiovascular abnormalities (e.g., hypotension, shock, and abnormal EKG), acute kidney injury, and dark brown urine, respectively [5]. Additional criteria for antivenom treatment include some local envenoming symptoms, such as severe local swelling, the rapid extension of swelling, and the development of an enlarged tender lymph node [5]. To prevent or minimise possible complications, such as infection, pain and inflammation, physicians prescribe antibiotics, tetanus toxoid, anti-inflammatory medications, and analgesic agents as adjuvants for snakebite treatment [9].

However, ineffective treatment and fatal outcomes following snakebite envenoming are reported in some rural areas of the world. These might be due to insufficient antivenom dosage or the use of a monospecific antivenom of inappropriate specificity [6,10]; delayed hospital treatment due to problems with transportation, inadequate ventilators or the failure to treat hypovolemic shock including infectious complications after hospitalisation [5,9]. In addition, antivenom administration potentially causes serious adverse reactions, such as anaphylaxis or serum sickness. Amid these problems, the mechanisms behind adverse reactions to snake antivenom remain poorly understood [11].

The incidence of snakebite envenoming is continuously decreasing in Thailand, but there are remaining problems, including early adverse reactions following snake antivenom administration [12] and disability and death due to severe infection [13]. Moreover, the early management and pharmacological treatment of adverse reactions following snake antivenom administration have been inadequately understood or practiced in some community hospitals [9]. To address these deficiencies, we investigated the factors associated with the aforementioned reactions via a retrospective analysis of data on snakebite envenoming victims in Narathiwat, the southernmost province of Thailand. Such association was determined based on the characteristics of snakebitten patients who received antivenom therapy, including demographic attributes, clinical symptoms, and laboratory results. Information regarding the prescription of antibiotics, NSAIDs, acetaminophen, tramadol, and premedications for preventing adverse reactions (i.e., antihistamine and epinephrine) was also evaluated. The findings can serve as a reference for the development of clinical guidelines on the administration and validation of snakebite treatment across the continent.

## 2. Results

### 2.1. Demographic Characteristics and Clinical Manifestations Following Snakebite Envenoming

**Figure 1:**
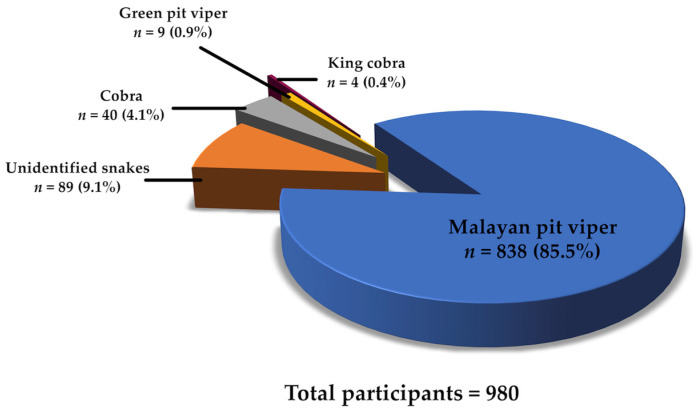
Total number of snakebite patients in five partner hospitals and classification of snake species.

There were 980 snakebite cases in our partner hospitals over the five-year study period. Among these, 89 patients were excluded because they were bitten by unknown snakes (Figure 1). A total of 513 Thai patients who were administered snake antivenom were included in this study. These patients were categorized into two groups based on their response to snake antivenom; the symptomatic group, comprising those with reported adverse reactions following antivenom administration (e.g., skin rash, angioedema, chest tightness and itching etc.), and asymptomatic group, consisting of patients who exhibited no such reactions following antivenom administration. The demographic characteristics of the patients are presented in Table 1. Males accounted for 72.1% of the participants, and more than half of them were between 26 and 59 years old (57.9%). Adverse reactions following snake antivenom administration were observed in 68 patients (symptomatic group: 13.3%). The Malayan pit viper was the leading cause of envenoming (95.7%), cobras were responsible for envenoming 17 patients (3.3%), and the king cobra and green pit viper caused envenomation in three (0.6%) and two (0.4%) cases, respectively. Among the patients, 87 were referred cases, and 25 (28.7%) exhibited significant adverse reactions after antivenom treatment (*p* < 0.01). Most of the victims were bitten on the foot (58.7%) and hand (25.9%). Clinical manifestations (Table 2) following snakebite envenoming included pain/swelling (71.3%), local bleeding (23.8%), ecchymosis (5.5%), bleb (1.8%), ptosis (1.2%), necrosis (0.2%), and respiratory failure (0.2%). One victim died (Table 1).

### 2.2. Laboratory Investigation Results

As shown in Table 3, the majority of the patients had normal levels of serum sodium (57.9%), serum potassium (47.6%), bicarbonate (37.6%), and chloride (40.4%). An increase in serum creatinine by ≥0.3 mg/dL within 48 h was observed in 15 patients. Platelet count testing was performed in 456 of the cases, with the test indicating that 47 (9.2%) cases exhibited low platelet counts (<100 × 10^3^ platelet/µL). The VCT test on 216 patients showed that 44 (8.6%) had prolonged VCT (>20 min). Prolonged INR (>1.2) was found in 43 patients (8.4%), and leucocytosis was detected in 178 cases (white blood cell count ≥11 × 10^3^ cells/µL, 34.7%). A significant increase in white blood cell count (≥11 × 10^3^ cells/µL) was found in the symptomatic group (15.7%, *p* = 0.0370).

### 2.3. Pharmacological and Nonpharmacological Treatments Following Snakebite Envenomation

**Figure 2:**
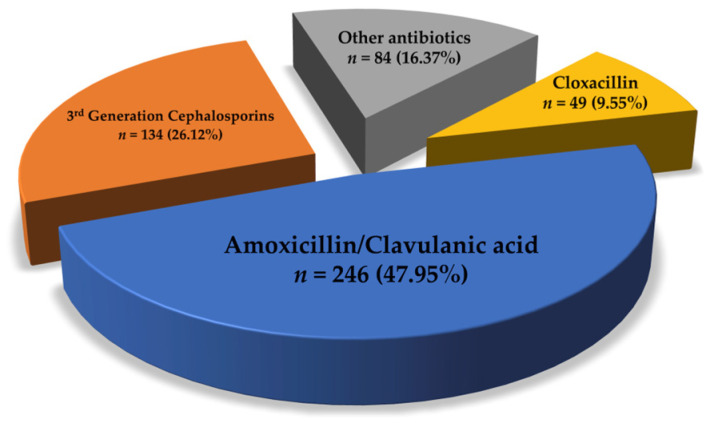
Antibiotics prescribed to receivers of antivenom therapy.

Antibiotics and acetaminophen were mostly prescribed to 495 (96.5%) and 468 patients (91.2%), respectively. For infection prevention, amoxicillin/clavulanic acid was the most frequently prescribed antibiotic (47.95%), while third-generation cephalosporins were administered to 134 patients (26.12%) (Figure 2). NSAIDs were administered to 43 patients after envenoming. Tramadol significantly reduced the occurrence of adverse reactions following antivenom administration in 273 patients (90.4%, *p* = 0.0040) (Table 4).

Nonpharmacological treatments, including wound debridement (1.8%), fasciotomy (1.4%), intubation (1.6%), and amputation (0.2%), were performed together with snake antivenom administration (Table 4).

### 2.4. Administration of Snake Antivenoms and the Occurrence of Adverse Reactions

**Figure 3:**
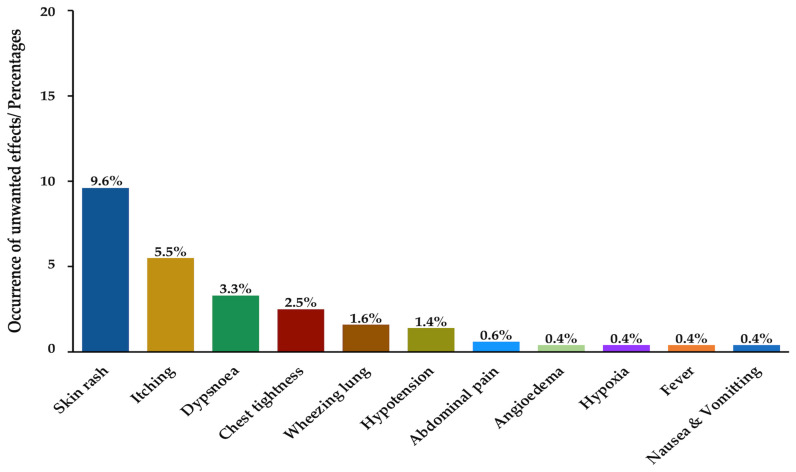
Occurrence of adverse reactions following the administration of QSMI snake antivenoms.

The administration of Malayan pit viper antivenom (≤5 vials) caused adverse reactions in 54 cases (12.9%). The administration of cobra monovalent (≤10 vials) and haemato-polyvalent (≤5 vials) antivenoms caused hypersensitivity symptoms (3.1% and 0.2%, respectively) (Table 5). Most of the antivenom recipients exhibited skin rashes (9.6%) and itching (5.5%) following antivenom administration, which also caused significant dyspnoea (3.3%) and chest tightness (2.5%) (*p* < 0.001). Other adverse reactions, including angioedema, wheezing lung, hypoxia, hypotension, fever, nausea/vomiting, and abdominal pain, were also reported (Figure 3, Table 6).

Epinephrine and antihistamines were prescribed in significant amounts to patients who presented with antivenom-induced adverse reactions (*p* < 0.001). Moreover, 85 asymptomatic patients were administered antibiotics following antivenom treatment (Table 5).

### 2.5. Factors Associated with Adverse Reactions

To evaluate the factors associated with adverse reactions following antivenom treatment, we subjected significant and likely determinants to multivariable analysis. These factors were age, gender, admission type, local bleeding, pain and swelling, laboratory factors (i.e., white blood cell count, VCT), the use of antihistamines, tramadol, antibiotics, and epinephrine. The factors derived after adjustment for potential confounders were admission type (referral vs. ER patients) and tramadol-induced reduction in adverse effects (Table 7). A multicollinearity test was performed to confirm that these two factors were independent of each other. Antihistamine use was significantly related to the reduction in adverse reactions following antivenom treatment, and epinephrine was prescribed significantly more frequently to the symptomatic group.

## 3. Discussion

The administration of snake antivenom is critical for the systemic treatment of snakebite envenoming. The QSMI of the Thai Red Cross Society is the only antivenom manufacturer producing snake F(ab′)2 antivenoms against medically important snakes in Thailand. In this study, four types of monospecific antivenoms and one polyvalent antivenom were administered to 513 snakebite envenoming patients. Snake antivenoms consist of neutralising antibodies produced in hyperimmunised animals (typically sheep or horses) and are highly effective products for the treatment of envenomation. However, their administration potentially causes life-threatening side effects [14], which can manifest as either early or late adverse reactions. Early adverse reactions are mostly severe and found within 24 h of antivenom administration, whereas late adverse reactions or serum sickness occur five to 20 days after antivenom treatment [11]. Early adverse reactions can be subdivided into (1) IgE-mediated anaphylactic reaction or type I hypersensitivity or immediate hypersensitivity, which involves the degranulation and release of histamines, prostaglandins, leukotrienes and other pharmacological mediators; (2) non-IgE-mediated anaphylactic reactions or anaphylactoid reactions caused by complement activation via IgG aggregates or the presence of Fc fragments or heterophilic antibodies against host cells, together with the stimulation of mast cells or basophils by antivenom proteins [15]; and (3) pyrogenic reactions, which are associated with endotoxin contamination (e.g., bacterial endotoxins) during antivenom production [16]. Fortunately, most antivenom production laboratories impose strict quality requirements on processing systems and equipment to avoid endotoxin contamination. Meanwhile, serum sickness is classified as a late adverse reaction and is activated by the humoral immunocomplex [17]. Antivenom reactions were previously attributed to the IgG immune complex or the contamination of antivenoms [14,15]. Nowadays, however, attribution to non-IgE factors appears to be more acceptable, as many studies, including the current research, have found that patients who develop adverse reactions can receive subsequent doses of the same antivenom without the occurrence of such reactions [12].

In this study, 13.3% of the patients who received antivenom treatment showed adverse reactions following the administration of snake antivenom manufactured by the QSMI. Referral patients exhibited significant adverse reactions after administration. These findings can be attributed to the severity of snakebite outcomes, the dosage of antivenom administered, and the method of administration employed. Some of the patients were referred from community or primary care units in Narathiwat, where there are limitations in antivenom, staff and healthcare equipment. Moreover, late adverse reactions might have stemmed from long hospitalisation. Symptoms related to anaphylactic reactions commonly occurred among 68 patients treated with antivenom. Cutaneous reactions (i.e., skin rash and itching) were typical side effects following the administration of all kinds of QSMI antivenoms [18]. Respiratory symptoms, such as wheezing lung and dyspnoea, were the second most common side effects in the respiratory tract. Pyrogenic adverse reactions, such as fever after QSMI antivenom administration, have rarely been reported given that pyrogenic testing is a routine quality control procedure implemented by manufacturers. Nevertheless, in this study, two fever cases were found following QSMI antivenom administration. In fact, low contamination is found in pharmaceutical products, but small amounts of endotoxins in antivenoms may cause a significant increase in fever incidence [19].

In Thailand, cobra (*N. kaouthia*), green pit viper (*T. albolabris*)*,* Russell’s viper (*D. siamensis*) and Malayan pit viper (*C. rhodostoma*) are classified as venomous snakes of category 1, which means that bites from these snakes result in high levels of morbidity and mortality [20,21]. Southern Thailand has the highest incidence of Malayan pit viper envenoming [22,23], and rubber tappers, a major occupation in the southern region, are the most common snakebite victims. A higher risk of snakebite is found among males than among females, especially in young adults, because of the agricultural activities in which they engage [24].

Snake identification is crucial for optimal clinical management, particularly when choosing a specific and appropriate snake antivenom. In this study, patients who were bitten by unknown snakes were excluded to minimise confounding factors. One patient died due to Malayan pit viper envenoming, but no evidence of antivenom-induced adverse reactions was found. Obtained data from registered nurse indicated that this case was a 12-year-old boy who received the Malayan pit viper antivenom due to the abnormal INR (1.33) following envenoming for 5 h.

Moreover, no association was found between adverse reactions and antivenoms or types of snakes in this study. The highest incidences of reaction were found for cobra envenoming (29.4%) and Malayan pit viper envenoming (12.8%). In fact, antivenoms against green pit viper and Malayan pit viper bites induce considerable adverse reactions [12,25]. This phenomenon might be due to the large populations of green pit viper and Malayan pit viper in the region of interest. Further investigations are needed to confirm the correlation between adverse reactions and types of venomous snakes. In addition, the types and dosages of snake antivenoms used were unrelated to adverse effects. Previous studies reported that cobra, Malayan pit viper and green pit viper antivenoms cause frequent and severe adverse reactions, which stem from the type of antivenom used, the method of administration and the dosage administered [25,26,27].

We did not record details regarding methods of antivenom administration, but Sriapha et al. (2022) and Holstege et al. (2002) found an association between the duration of antivenom administration and the occurrence of adverse reactions [12,28]. They also showed that an infusion time of 30 to 60 min significantly attenuates the incidence of adverse reactions [12]. As most patients exhibit adverse reactions within 1 h after the completion of antivenom administration, the continuous monitoring of such reactions is required at least 2 h after the initiation of antivenom infusion [12].

The laboratory investigations showed that an increase in white blood count higher than 11 × 10^3^ cells/µL was significantly associated with the presence of adverse reactions following antivenom administration. Inflammation, infection, malignancy, and allergic reactions are common causes of leucocytosis. Leucocytosis is frequently observed in snakebite envenoming patients and, more generally, in patients receiving antivenom therapy [29]. Antihistamines and epinephrine were prescribed to 117 and 13 patients, respectively. In many countries, premedications are administered to prevent snake antivenom-induced adverse reactions [17]. Glucocorticoids and antihistamines are commonly used, but epinephrine administration is rare [14,30]. Nevertheless, premedication as a preventative is controversial given its low efficacy [14]. We found that antihistamines were significantly more frequently prescribed to the asymptomatic patients (72.6%) than to the symptomatic group (27.4%), suggesting that antihistamines were regarded as premedications. The main outcomes triggered by histamines include direct vasodilation involving oedema, reduced blood pressure, bronchoconstriction, urticaria, and itching [31]. H1 antihistaminic agents, especially first-generation H1 antagonists such as promethazine and chlorpheniramine, are the drugs of choice for preventing or treating allergic reactions [32]. Epinephrine appears to be the most widely used catecholamine for the treatment of anaphylactic reactions due to its strong and extensive effects [33]. In the current work, the 61.5% of patients administered epinephrine showed adverse reactions, suggesting that epinephrine administration serves only as treatment for acute adverse reactions and not pretreatment. Moreover, tramadol administration resulted in significantly fewer adverse reactions. This medication has an inhibitory effect on histamine release, unlike codeine, pethidine, and morphine, which have the greatest histamine-releasing capacity [34].

Other pharmacological treatments, including acetaminophen, NSAIDs and antibiotics, had no significant association with adverse reactions. Envenoming-related tissue injuries are significant in microbial proliferation, causing tissue damage. Snakebite-related infections have also been ascribed to *Morganella morganii*, *Enterococcus* spp., *Staphylococcus aureus*, and anaerobes [35]. Finally, the effectiveness of prophylactic antimicrobial use promotes bacterial resistance [36,37,38]. Thus, the rational use of antimicrobials is necessary during initial empirical treatment, along with the isolation and identification of bacteria that may be present in snakebite wounds [35]. In the current study, we found that amoxicillin/clavulanic acid was the most frequently prescribed antibiotic for snakebite envenoming victims. However, piperacillin/tazobactam, ciprofloxacin and third-generation cephalosporins are the most appropriate antibiotics for empiric therapy. Amoxicillin/clavulanic acid is recommended as preventatives for animal bites, particularly those from cats, dogs and humans, but not snakes [39,40]. That is, amoxicillin/clavulanic acid is ineffective against the bacteria present in a snake’s mouth [41].

Given that this was a retrospective study which corrected by the healthcare professionals in provincial hospitals, it was encumbered by certain limitations, such as missing data from the laboratory investigations and diagnoses. Information on clinical manifestations was collected by primary clinicians and nurses. The quality of conclusions drawn may have been diminished given the small sample of snakebite envenomed patients, and this research was conducted in only one province in southern Thailand. Therefore, the data may not be generalisable to other regions.

## 4. Conclusions

Snakebite envenoming is a devastating environmental and occupational disease that results in high levels of fatality and disability in many rural areas. In this study, adverse reactions occurred in 13.3% of the patients following snake antivenom administration—a phenomenon that tends to occur among referral patients. This means that the reactions may have been caused by prior relevant treatments and individual immune systems. The dosages and types of administered snake antivenoms, as well as the use of antibiotics, NSAIDs and acetaminophen, had no significant relationship with hypersensitivity. Patients who were administered tramadol or antihistamine showed a significant decrease in adverse reactions following antivenom treatment. Further studies are required to elucidate the factors and responsible mechanisms behind antivenom-induced adverse reactions. Close monitoring alongside resuscitation equipment and medications that minimise the severity of anaphylactic reactions are necessary. This study was conducted under the holistic idea that the clinical treatment of snakebite envenomation should involve not only antivenom therapy but also pharmacological treatments and treatment of adverse reactions. The findings are expected to serve as a resource for the development of clinical guidelines for treating snakebite envenoming and the validation of snakebite treatment across the continent.

## 5. Materials and Methods

### 5.1. Study Design and Subjects

This study was of a retrospective descriptive design involving the collection of data on snakebitten patients from 1 November 2016 to 31 October 2021. Five participating hospitals were chosen as previously described [6]. Briefly, the patients included in the study were those admitted to Ra-Ngae Hospital (120 beds), Rueso Hospital (90 beds), Yi-ngo Hospital 80th Anniversary Commemoration Hospital (60 beds), Takbai Hospital (120 beds), and Naradhiwas Rajanagarindra Hospital (400 beds), which are located in Narathiwat Province. The inclusion criteria were snakebitten envenoming patients who were administered antivenom during hospital admission and patients bitten by the Malayan pit viper, cobra, King cobra, and green pit viper, as determined through (1) the snake carcasses brought to the hospital by the patients and (2) the clear descriptions or identification of the types of snakes that bit them. Patients with snakebites from unknown species were excluded. For data analysis, the eligible patients were categorised based on the presence of adverse reactions into the overall group, the symptomatic group, and the asymptomatic group (Supplementary Material File S1).

### 5.2. Data Collection

At each hospital, a well-trained registered nurse abstracted medical records using a standardised case report form (CRF) verified by a team of senior researchers. Snakebites were identified using code T63.0 of the International Classification of Diseases (Tenth Revision), as documented in the medical records. Of these, only identified snakebite envenoming patients were included in the research. The CRF data were then converted into electronic form via documentation on a spreadsheet. The collected data comprised demographic characteristics; snake types; types of snake antivenoms; the presence of symptoms following antivenom administration; bite areas; fang marks; local effects, including pain, swelling, local bleeding, ecchymosis, bleb, necrosis and impending compartment syndrome; systemic effects, including prolonged VCT, unclotted 20WBCT, an INR > 1.2, thrombocytopenia and systemic bleeding; and other laboratory investigation parameters, such as electrolytes, creatine phosphokinase, creatinine and white blood cell count. Local and systemic effects were recorded at the start of admission and during hospital stay. All laboratory investigations were performed prior to treatment. The management of adverse reactions following antivenom administration (i.e., the prescription of epinephrine, corticosteroids, inotropic agents, and antihistamines) was recorded. Other pharmacological and nonpharmacological treatments were also included in the evaluation of factors associated with antivenom-induced adverse reactions, such as the prescription of antibiotics, tramadol, NSAIDs, and tetanus toxoid.

### 5.3. Statistical Analyses

The data were analysed using Stata Statistical Software: Release 17 (StataCorp LLC., College Station, TX, USA). Demographic characteristics were examined via descriptive statistical analysis. The outcomes were presented as numbers and percentages for the categorical data and as medians, interquartile ranges, and ranges (minimum to maximum) for the continuous data. Proportions were calculated proportionally to the total number of each data group obtained. The Mann–Whitney U test was conducted to verify differences in medians, while the chi-square or Fisher’s exact test was carried out to compare differences in the distribution proportion of categorical variables between patients who presented with adverse reactions following antivenom therapy and patients who had no such reactions. Univariate and multivariate logistic regression analyses were performed to determine the factors associated with adverse effects. Age, gender, pain and swelling and VCT were included in the univariate logistic regression analyses. Admission type, white blood cell counts and receipt of epinephrine, antihistamines, antibiotics, and tramadol were adjusted in the final model. The magnitudes of associations obtained from the univariate and multivariate analyses were represented as crude odds ratios and adjusted odds ratios, respectively, with their corresponding 95% confidence intervals (CIs). Statistical significance was set at *p*  < 0.05.

### 5.4. Ethical Considerations

This study was reviewed and approved by the Institutional Review Board of the Royal Thai Army Medical Department, Bangkok, Thailand (project code: S097h/64_Exp, last approval date 26 December 2024). This study was conducted in accordance with the principles of the Declaration of Helsinki. The requirement for informed consent was waived by the Institutional Review Board because this is a retrospective study of deidentified data retrieved from medical records.

## Figures and Tables

**Table 1:** Demographic characteristics of patients and toxic outcomes following snakebite.

Characteristics	Asymptomatic Group *n* = 445		Symptomatic Group *n* = 68		*p*-Value	Overall *n* = 513	
	*n* (% of Row Item)	Proportions	*n* (% of Row Item)	Proportions		*n* (% of 513 Patients)	Proportions
**Sex**					0.669		
Male	322 (87.0)	0.72	48 (13.0)	0.71		370 (72.1)	0.72
Female	123 (86.0)	0.28	20 (14.0)	0.29		143 (27.9)	0.28
**Age (years)**					0.602		
0–25	103 (87.3)	0.23	15 (12.7)	0.22		118 (23.0)	0.23
26–59	259 (87.7)	0.59	38 (12.3)	0.56		297 (57.9)	0.58
>60	77 (83.7)	0.18	15 (16.3)	0.22		92 (17.9)	0.18
**Snake Type**					0.195		
Cobra	12 (70.6)	0.03	5 (29.4)	0.07		17 (3.3)	0.03
King cobra	3 (100)	0.01	0 (0.0)	0.00		3 (0.6)	0.01
Malayan pit viper	428 (87.2)	0.96	63 (12.8)	0.93		491 (95.7)	0.96
Green pit viper	2 (100)	0.00	0 (0.0)	0.00		2 (0.4)	0.00
**Admission Type**							
Referral	62 (71.3)	0.14	25 (28.7)	0.37	<0.01 *	87 (17.0)	0.17
ER	383 (89.9)	0.86	43 (10.1)	0.63		426 (83.0)	0.83
**Fang mark**					0.721		
Absence	46 (85.2)	0.10	8 (14.8)	0.12		54 (10.5)	0.11
Presence	399 (86.9)	0.90	60 (13.1)	0.88		459 (89.5)	0.89
**Bitten area**					0.857		
Arm	4 (100)	0.01	0 (0.0)	0.00		4 (0.8)	0.01
Hand	119 (89.5)	0.27	14 (10.5)	0.21		133 (25.9)	0.26
Leg	49 (86.0)	0.11	8 (14.0)	0.12		57 (11.1)	0.11
Foot	258 (85.7)	0.58	43 (14.3)	0.64		301 (58.7)	0.59
Others	9 (90.0)	0.02	1 (10.0)	0.01		10 (1.9)	0.02
Unidentified	5 (83.3)	0.01	1 (16.7)	0.01		6 (1.2)	0.01
**Death**					NA		
No	444 (86.7)	0.99	68 (13.3)	1.00		512 (99.8)	1.0
Yes	1 (100)	0.00	0 (0.0)	0.00		1 (0.2)	0.0

NA = not available. * is significantly different.

**Table 2:** Clinical manifestations following snake envenoming.

Characteristics	Asymptomatic Group *n* = 445		Symptomatic Group *n* = 68		*p*-Value	Overall *n* = 513	
	*n* (% of Row Item)	Proportions	*n* (% of Row Item)	Proportions		*n* (% of 513 Patients)	Proportions
**Ecchymosis**					0.460		
No	422 (87.0)	0.95	63 (13.0)	0.926		485 (94.5)	0.95
Yes	23 (82.1)	0.05	5 (17.9)	0.074		28 (5.5)	0.05
**Local bleeding**					0.059		
No	333 (85.2)	0.75	58 (14.8)	0.85		391 (76.2)	0.76
Yes	112 (91.8)	0.25	10 (8.2)	0.15		122 (23.8)	0.24
**Ptosis**					0.145		
No	441 (87.0)	0.99	66(13.0)	0.97		507 (98.8)	0.99
Yes	4 (66.7)	0.01	2 (33.3)	0.03		6 (1.2)	0.01
**Bleb**					0.423		
No	438 (86.9)	0.98	66 (13.1)	0.97		504 (98.2)	0.98
Yes	7 (77.8)	0.02	2 (22.2)	0.03		9 (1.8)	0.02
**Necrosis**					NA		
No	445 (86.9)	1.00	67 (13.1)	0.99		512 (99.8)	1.00
Yes	0 (0.0)	0.00	1 (100)	0.01		1 (0.2)	0.00
**Pain and swelling**					<0.05 *		
No	135 (91.8)	0.30	12 (8.2)	0.18		147 (28.7)	0.29
Yes	310 (84.7)	0.70	56 (15.3)	0.82		366 (71.3)	0.71
**Dark brown urine**					NA		
No	445 (86.7)	1.00	68 (13.3)	1.00		513 (100)	1.0
Yes	0 (0.0)	0.00	0 (0.0)	0.00		0 (0.0)	0.0
**Respiratory failure**					<0.001 *		
No	445 (86.9)	1.00	67 (13.1)	0.99		512 (99.8)	1.00
Yes	0 (0.0)	0.00	1 (100)	0.01		1 (0.2)	0.00

NA = not available. “*” is significantly different.

**Table 3:** Laboratory investigation results for receivers of snake antivenom treatment.

Laboratory Investigations	Asymptomatic Group *n* = 445		Symptomatic Group *n* = 68		*p*-Value	Overall *n* = 513	
	*n* (% of Row Item)	Proportions	*n* (% of Row Item)	Proportions		*n* (% of 513 Patients)	Proportions
**Sodium (mEq/L)**					0.411		
<135	12 (100)	0.03	0 (0.0)	0.00		12 (2.3)	0.02
135–145	261 (87.9)	0.59	36 (12.1)	0.53		297 (57.9)	0.58
>145	5 (83.3)	0.01	1 (16.7)	0.01		6 (1.2)	0.01
**Potassium (mEq/L)**					0.929		
<3.5	64 (87.7)	0.14	9 (12.3)	0.13		73 (14.2)	0.14
3.5–5.5	215 (88.1)	0.48	29 (11.9)	0.43		244 (47.6)	0.48
>5.5	1 (100)	0.00	0 (0.0)	0.00		1 (0.2)	0.00
**Chloride (mEq/L)**					0.812		
<90	1 (100)	0.00	0 (0.0)	0.00		1 (0.2)	0.00
90–105	183 (88.4)	0.41	24 (11.6)	0.35		207 (40.4)	0.40
>105	75 (86.2)	0.17	12 (13.8)	0.18		87 (17.0)	0.17
**Bicarbonate (mEq/L)**					0.393		
<20	10 (76.9)	0.02	3 (23.1)	0.04		13 (2.5)	0.03
20–26	168 (87.0)	0.38	25 (13.0)	0.37		193 (37.6)	0.38
>26	73 (90.1)	0.16	8 (9.9)	0.12		81 (15.8)	0.16
**Creatine phosphokinase (units/L)**					0.248		
≤1 × 10^3^	0 (0.0)	0.00	1 (100)	0.02		1 (0.2)	0.00
>1 × 10^3^	2 (66.7)	0.00	1 (33.3)	0.02		3 (0.6)	0.01
**Creatinine at admission**					0.608		
No	2 (100)	0.00	0 (0.0)	0.00		2 (0.4)	0.00
Yes	266 (88.4)	0.99	35 (11.6)	0.51		301 (58.7)	0.59
**Creatinine rising ≥ 0.3 mg/dL (within 48 h)**					NA		
No	0 (0.0)	0.00	0 (0.0)	0.00		0 (0.0)	0.00
Yes	12 (80.0)	0.03	3 (20.0)	0.04		15 (2.9)	0.03
**White blood cell count (cells/µL)**					0.037 *		
<11 × 10^3^	254 (90.7)	0.57	26 (9.3)	0.38		280 (54.6)	0.55
≥11 × 10^3^	150 (84.3)	0.34	28 (15.7)	0.41		178 (34.7)	0.35
**VCT (min)**					0.166		
≤20	152 (88.4)	0.34	20 (11.6)	0.29		172 (33.5)	0.34
>20	42 (95.5)	0.09	2 (4.5)	0.03		44 (8.6)	0.09
**INR**					0.695		
≤1.2	64 (73.6)	0.14	23 (26.4)	0.34		87 (17.0)	0.17
>1.2	33 (76.7)	0.07	10 (23.3)	0.15		43 (8.4)	0.08
**Platelet count (platelets/µL)**					0.824		
<100 × 10^3^	42 (89.4)	0.09	5 (10.6)	0.07		47 (9.2)	0.09
≥100 × 10^3^	361 (88.3)	0.81	48 (11.7)	0.71		409 (79.7)	0.80

NA = not available. “*” is significantly different.

**Table 4:** Pharmacological and nonpharmacological treatments following snakebite.

Laboratory Investigations	Asymptomatic Group *n* = 445		Symptomatic Group *n* = 68		*p*-Value	Overall *n* = 513	
	*n* (% of Row Item)	Proportions	*n* (% of Row Item)	Proportions		*n* (% of 513 Patients)	Proportions
**Pharmacological Treatments**							
**NSAIDs**					0.205		
No	405 (86.2)	0.91	65 (13.8)	0.96		470 (91.6)	0.92
Yes	40 (93.0)	0.09	3 (7.0)	0.04		43 (8.4)	0.08
**Acetaminophen**					0.063		
No	35 (77.8)	0.08	10 (22.2)	0.15		45 (8.8)	0.09
Yes	410 (87.6)	0.92	58 (12.4)	0.85		468 (91.2)	0.91
**Tramadol**					0.004 *		
No	172 (81.5)	0.39	39 (1.5)	0.57		211 (41.1)	0.41
Yes	273 (90.4)	0.61	29 (9.6)	0.43		302 (58.9)	0.59
**Tetanus toxoid**					0.349		
No	395 (87.2)	0.89	58 (12.8)	0.85		453 (88.3)	0.88
Yes	48 (82.8)	0.11	10 (17.2)	0.15		58 (11.3)	0.11
**Antibiotics**					0.253		
No	14 (77.8)	0.03	4 (22.2)	0.06		18 (3.5)	0.04
Yes	431 (87.1)	0.97	64 (12.9)	0.94		495 (96.5)	0.96
**Non-pharmacological treatments**							
**Wound Debridement**					0.848		
No	437 (86.7)	0.98	67 (13.3)	0.99		504 (98.3)	0.98
Yes	8 (88.9)	0.02	1 (11.1)	0.01		9 (1.8)	0.02
**Fasciotomy**					0.935		
No	439 (86.8)	0.99	67 (13.2)	0.99		506 (98.6)	0.99
Yes	6 (85.7)	0.01	1 (14.3)	0.01		7 (1.4)	0.01
**Intubation**					0.323		
No	439 (86.9)	0.99	66 (13.1)	0.97		505 (98.4)	0.98
Yes	6 (75.0)	0.01	2 (25.0)	0.03		8 (1.6)	0.02
**Amputation**					NA		
No	445 (86.9)	1.00	67 (13.1)	0.99		512 (99.8)	1.00
Yes	0 (0.0)	0.00	1 (100)	0.01		1 (0.2)	0.00

NA = not available. “*” is significantly different.

**Table 5:** Type of Antivenom and pharmacological treatment for adverse reactions following antivenom administration.

Laboratory Investigations	Asymptomatic Group *n* = 445		Symptomatic Group *n* = 68		*p*-Value	Overall *n* = 513	
	*n* (% of Row Item)	Proportions	*n* (% of Row Item)	Proportions		*n* (% of 513 Patients)	Proportions
**Type of antivenom**							
**Malayan pit viper antivenom**					0.471		
≤5 vials	364 (87.1)	0.82	54 (12.9)	0.79		418 (81.5)	0.81
>5 vials	56 (90.3)	0.13	6 (9.7)	0.09		62 (12.1)	0.12
**Cobra antivenom**					NA		
≤10 vials	12 (75.0)	0.03	4 (25.0)	0.06		16 (3.1)	0.03
>10 vials	0 (0.0)	0.00	0 (0.0)	0.00		0 (0.0)	0.00
**King cobra antivenom**					NA		
≤10 vials	2 (100)	0.00	0 (0.0)	0.00		2 (0.4)	0.00
>10 vials	0 (0.0)	0.00	0 (0.0)	0.00		0 (0.0)	0.00
**Green pit viper antivenom**					NA		
≤3 vials	2 (100)	0.00	0 (0.0)	0.00		2 (0.4)	0.00
>3 vials	0 (0.0)	0.00	0 (0.0)	0.00		0 (0.0)	0.00
**Hemato-polyvalent antivenom**					NA		
≤5 vials	0 (0.0)	0.00	1 (100)	0.01		1 (0.2)	0.00
>5 vials	1 (100)	0.00	0 (0.0)	0.00		1 (0.2)	0.00
**Treatments**							
**Epinephrine**					<0.001 *		
No	440 (88.0)	0.99	60 (12.0)	0.88		500 (97.5)	0.97
Yes	5 (38.5)	0.01	8 (61.5)	0.12		13 (2.5)	0.03
**Antihistamine**					<0.001 *		
No	360 (90.9)	0.81	36 (9.1)	0.53		396 (77.2)	0.77
Yes	85 (72.6)	0.19	32 (27.4)	0.47		117 (22.8)	0.23
**Inotropic drugs**					NA		
No	445 (87.9)	1.00	61 (12.1)	0.90		506 (98.6)	0.99
Yes	0 (0.0)	0.00	7 (100)	0.10		7 (1.4)	0.01

NA = not available. “*” is significantly different.

**Table 6:** Adverse reactions following antivenom administration.

Laboratory Investigations	Asymptomatic Group *n* = 445		Symptomatic Group *n* = 68		*p*-Value	Overall *n* = 513	
	*n* (% of Row Item)	Proportions	*n* (% of Row Item)	Proportions		*n* (% of 513 Patients)	Proportions
**Adverse reactions**							
**Skin rash**					<0.001 *		
No	445 (95.9)	1.00	19 (4.1)	0.28		464 (90.5)	0.90
Yes	0 (0.0)	0.00	49 (100)	0.72		49 (9.6)	0.10
**Itching**					<0.001 *		
No	445 (91.8)	1.00	40 (8.2)	0.59		485 (94.5)	0.95
Yes	0 (0.0)	0.00	28 (100)	0.41		28 (5.5)	0.05
**Angioedema**					NA		
No	445 (87.1)	1.00	66 (12.9)	0.97		511 (99.6)	1.00
Yes	0 (0.0)	0.00	2 (100)	0.03		2 (0.4)	0.00
**Wheezing lung**					NA		
No	445 (88.1)	1.00	60 (11.9)	0.88		505 (98.4)	0.98
Yes	0 (0.0)	0.00	8 (100)	0.12		8 (1.6)	0.02
**Dyspnoea**					<0.001 *		
No	445 (89.7)	1.00	51 (10.3)	0.75		496 (96.7)	0.97
Yes	0 (0.0)	0.00	17 (100)	0.25		17 (3.3)	0.03
**Chest tightness**					<0.001 *		
No	445 (89.0)	1.00	55 (11.0)	0.81		500 (97.5)	0.97
Yes	0 (0.0)	0.00	13 (100)	0.19		13 (2.5)	0.03
**Hypoxia**					NA		
No	445 (87.1)	1.00	66 (12.9)	0.97		511 (99.6)	1.00
Yes	0 (0.0)	0.00	2 (100)	0.03		2 (0.4)	0.00
**Hypotension**					NA		
No	445 (87.9)	1.00	61 (12.1)	0.90		506 (98.6)	0.99
Yes	0 (0.0)	0.00	7 (100)	0.10		7 (1.4)	0.01
**Fever**					NA		
No	445 (87.1)	1.00	66 (12.9)	0.97		511 (99.6)	1.00
Yes	0 (0.0)	0.00	2 (100)	0.03		2 (0.4)	0.00
**Nausea and Vomiting**					NA		
No	445 (87.1)	1.00	66 (12.9)	0.97		511 (99.6)	1.00
Yes	0 (0.0)	0.00	2 (100)	0.03		2 (0.4)	0.00

NA = not available. “*” is significantly different.

**Table 7:** Univariable and multivariable analyses of factors associated with antivenom-induced adverse reactions.

Factors	Adverse Reactions									
	Asymptomatic		Symptomatic		Univariate Analysis			Multivariable Analysis		
	*n*	%	*n*	%	*p*-Value	Crude Odds Ratio	95%CI	*p*-Value	Adjusted Odds Ratio	95%CI
**Age**										
0–25	103	87.3	15	12.7		1			1	
26–59	257	87.7	36	12.3	0.906	0.962	0.505–1.832	0.469	0.765	0.371–1.578
>60	77	83.7	15	16.3	0.461	1.338	0.617–2.901	0.904	1.058	0.423–2.648
**Gender**										
Male	322	87.0	48	13.0		1				
female	123	86.0	20	14.0	0.669	1.131	0.643–1.989			
**Admission Type**										
Referral	62	71.3	25	28.7	<0.001 *	3.592	2.049–6.295	≤0.001 *	3.078	1.576–6.012
ER	383	89.9	43	10.1		1				
**Pain and swelling**										
No	135	91.8	12	8.2		1				
Yes	310	84.7	56	15.3	0.034	2.032	1.055–3.914			
**Epinephrine**										
No	440	88.0	60	12.0		1			1	
Yes	5	38.5	8	61.5	<0.001 *	11.733	3.717–37.036	<0.05 *	6.634	1.379–31.92
**Antihistamine**										
No	360	90.9	36	9.1		1			1	
Yes	85	72.6	32	27.4	<0.001 *	3.765	2.212–6.406	<0.001 *	4.924	2.223–10.906
**Tramadol**										
No	172	81.5	39	18.5		1			1	
Yes	273	90.4	29	9.6	0.004 *	0.468	0.279–0.786	<0.05 *	0.456	0.243–0.857
**Antibiotics**										
No	14	77.8	4	22.2		1			1	
Yes	431	87.1	64	12.9	0.261	0.520	0.166–1.628	0.607	0.686	0.164–2.878
**White blood cell count (cells/µL)**										
<11 × 10^3^	254	90.7	26	9.3		1			1	
≥11 × 10^3^	150	84.3	28	15.7	0.039 *	1.824	1.031–3.227	0.169	1.569	0.826–2.98
**VCT (min)**										
<20	152	88.4	20	11.6	0.182	2.763	0.621–12.299			
≥20	42	95.5	2	4.5		1				

“*” is significantly different.

## Data Availability

The data presented in this study are available in this article or Supplementary Materials here.

## Supplementary Materials

The following supporting information can be downloaded at: https://www.mdpi.com/article/10.3390/toxins18030139/s1, Supplementary Material File S1.

## Abbreviations

The following abbreviations are used in this manuscript:

VCT	Venous clotting time
20WBCT	20 min whole blood clotting time
INR	international normalised ratio
QSMI	the Queen Saovabha Memorial Institute
